# Losing the Self in Near-Death Experiences: The Experience of Ego-Dissolution

**DOI:** 10.3390/brainsci11070929

**Published:** 2021-07-14

**Authors:** Charlotte Martial, Géraldine Fontaine, Olivia Gosseries, Robin Carhart-Harris, Christopher Timmermann, Steven Laureys, Héléna Cassol

**Affiliations:** 1 Coma Science Group, GIGA-Consciousness, University of Liège, 4000 Liège, Belgium; cmartial@uliege.be (C.M.); geraldine.fontaine@ulb.be (G.F.); ogosseries@uliege.be (O.G.); steven.laureys@uliege.be (S.L.); 2 Centre du Cerveau, University Hospital of Liège, 4000 Liège, Belgium; 3 Centre for Psychedelic Research, Division of Psychiatry, Imperial College London, London W12 0NN, UK; r.carhart-harris@imperial.ac.uk (R.C.-H.); christimmer@gmail.com (C.T.)

**Keywords:** near-death experience, ego dissolution, out-of-body experience, self, experiencer, nature-relatedness

## Abstract

Many people who have had a near-death experience (NDE) describe, as part of it, a disturbed sense of having a “distinct self”. However, no empirical studies have been conducted to explore the frequency or intensity of these effects. We surveyed 100 NDE experiencers (Near-Death-Experience Content [NDE-C] scale total score ≥27/80). Eighty participants had their NDEs in life-threatening situations and 20 had theirs not related to life-threatening situations. Participants completed the Ego-Dissolution Inventory (EDI) and the Ego-Inflation Inventory (EII) to assess the experience of ego dissolution and inflation potentially experienced during their NDE, respectively. They also completed the Nature-Relatedness Scale (NR-6) which measures the trait-like construct of one’s self-identification with nature. Based on prior hypotheses, ratings of specific NDE-C items pertaining to out-of-body experiences and a sense of unity were used for correlational analyses. We found higher EDI total scores compared with EII total scores in our sample. Total scores of the NDE-C scale were positively correlated with EDI total scores and, although less strongly, the EII and NR-6 scores. EDI total scores were also positively correlated with the intensity of OBE and a sense of unity. This study suggests that the experience of dissolved ego-boundaries is a common feature of NDEs.

## 1. Introduction

[“*Without aspiration or projection I crossed the tunnel at full speed. Speed is not the right word because there was no movement. It was more like a dissolution of myself and an equally sudden eclosion. Somehow, a lightning crossing. In short, I found myself split into two parts. My body was resting on the bed and from the top of a cloud I could see myself. My double on the bedroom ceiling was witnessing an extraordinary scene in sharpness and authenticity.*” Translated verbatim from French]

This verbatim extract, drawn from our collection of testimonies of near-death experiences (NDEs), includes mention of a dissolved sense of self, evoking comparisons with loss of self induced via other means such as meditation, sleep, or psychedelic drugs (e.g., [1,2,3]). NDEs are episodes of disconnected consciousness in which the person experiences various prototypical mental events, with highly emotional and mystical aspects [4]. NDEs typically occur in truly life-threatening situations such as cardiac arrest, traumatic injury, intracerebral hemorrhage, nearly drowning, or asphyxia [5]. NDEs can also occur in situations that feel life-threatening (e.g., high anxiety) and perhaps in situations such as meditation or sleep, which are then referred as “NDE-like” since the phenomenology is similar to a classical NDE yet without an actual imminent risk of death [6]. Currently, in research, the identification of NDE(-like) experiencers is based on the richness of the experience—that is, the presence and intensity of a number of defining features. The Near-Death Experience Content (NDE-C) scale has been very recently developed and validated to accurately identify these features and facilitate empirical research [7].

Among the defining features of NDEs, out-of-body experience (OBE) is the second most frequently reported feature, after a feeling of profound peacefulness [6,8,9,10]. OBEs are related to a compromised sense of possessing a single, integrated, and distinct identity, typically involving a shift in the subjectively experienced location of the perceived self and a disturbance of the sense of self (see [11] for a review). The construct of “the self” or “ego” is highly abstract and thus eludes a singular concrete definition (although, see these attempts: [12,13]), but it is often thought of as a subjective perception of oneself as a distinct entity [14] combined with a sense of a ‘narrative self’ or identity [15,16]. A disturbance of the sense of self may take various forms, ranging from a small disturbance of self-perception to completely losing the sense of ego.

In 2000, Greyson [17] found that NDE experiencers easily or frequently experience common and non-pathological dissociation states before and/or after having had their NDE, such as feeling as if their body does not belong to them. Such dissociative symptoms inherently include a disturbance of the sense of self by the partial or complete loss of the normal coordination between self, body, and environment [18]. However, so far, no empirical study has rigorously investigated the sense of self during the NDE itself, even though most experiencers seem to subsequently report a disruption of their sense of self when describing their experience.

The disturbance of the sense of self, in the form of an OBE or in any other form, may arise in many other types of contexts than during classical NDEs: it can occur spontaneously or in a specific pathological (e.g., acute psychosis; [19]) or non-pathological condition (e.g., meditation, sleep or drug-induced psychedelic experiences; [1,2,3]). Notably, evidence from studies involving psychedelic drugs has repeatedly shown that the experience of ego dissolution (i.e., a disruption of ego-boundaries which leads to a partial or complete blurring of the distinction between the self and the rest of the environment and other objects or people combined with a loss of self-identity or ‘narrative self’) is a very common feature of drug-induced experiences, especially following the ingestion of high doses [12,20]. This is especially true for classic psychedelic drugs (i.e., 5-HT2A receptor agonists) such as psilocybin [21,22] and N, N-Dimethyltryptamine (DMT) [23], as well as dissociative anesthetics such as ketamine [24], and the terpene salvinorin–A [25]. Specifically, many psychedelic drug ‘experience reports’ reference a sense of continuity or unity between their self and the external world [3], evoking the notion of disturbed ‘ego-boundaries’ [26] and the ‘unitive experience’ (defined by [27] as a sense of all-encompassing ‘unity’ or ‘oneness’). Importantly, the intensity of ego dissolution experienced under psychedelics correlates with the subsequently reported feeling of connectedness with nature and humanity [13,28,29]. Recently, Nour and colleagues [3] developed the Ego-Dissolution Inventory (EDI), a standardized scale designed to capture the experience of ego dissolution and associated feeling of unity with one’s surroundings. Its development was based on the ratings of 691 participants linked to their subjective experiences under classical psychedelics. The final scale includes eight items related to ego dissolution. In parallel, to aid the validation process, the authors also developed eight other items to assess the experience of ‘ego inflation’, which corresponds to an “unusually elevated self-assuredness and confidence” [3]. In short, they demonstrated that the experience of ego dissolution positively correlated with the dose and intensity of effects of a psychedelic drug, whereas the reported dosage of a control drug, cocaine, correlated with ego-inflation scores. Validation of the EDI and Ego-Inflation Inventory (EII) suggested a distinct factor structure separating the two measures but it was not clear if they were inversely related as one might assume. Interestingly, recent studies have shown that NDE-like episodes can be induced by the classic serotonergic psychedelic DMT [23] or ketamine [30].

So far, no study has explored the sense of ego dissolution and inflation in a large sample of people who have experienced an NDE. In the present work, we sought to study the experience of ego dissolution and inflation in people who reported a classical NDE or NDE-like episode. To do this, we retrospectively administered the following questionnaires to assess their experience itself: (i) the NDE-C [7] to quantify the NDE(-like) phenomenology, (ii) the EDI [3] to explore the sense of ego dissolution potentially experienced during the NDE(-like), and (iii) the EII [3] to assess the experience of an ego inflation potentially experienced during the NDE(-like). Finally, we administered (iv) the Nature-Relatedness Scale (NR-6; [31]) to evaluate the participants’ potential subjective sense of connectedness with nature that they experienced at the time of the study. This trait-like construct was thus measured retrospectively to their NDE. Since we cannot currently distinguish between classical NDEs and NDE-like episodes (i.e., the studies conducted so far did not find differences in terms of intensity or frequency of core features when comparing both groups; e.g., [6,7]), we did not expect significant differences between classical NDE experiencers’ and NDE-like experiencers’ responses to these questionnaires. However, given that very few empirical articles include a group of NDE-like experiencers and that these experiences emerge in distinct contexts, we decided to examine differences between both subgroups of participants. We predicted NDE experiencers to score higher on the EDI than on the EII and positive correlations of the self-reported richness of NDEs (i.e., NDE-C scale total score) with the experience of ego dissolution (i.e., EDI total score) and connectedness with nature (i.e., NR-6 total score). Finally, we explored the relationship of two NDE features associated with ego dissolution, namely OBEs and experiences of unity with a larger whole. OBE and unity are two specific items contained within the NDE-C.

## 2. Materials and Methods

### 2.1. Participants and Procedure

Participants were recruited among people who contacted us to share their experiences. Initially, they were recruited through websites, social media, appearances in local news and publications of the Coma Science Group (GIGA-Consciousness, University of Liège, Belgium). They were mailed questionnaires including questions related to socio-demographic (i.e., gender, age at interview, age at NDE, time since NDE) and clinical (i.e., precipitating factors, presence of a life-threatening event) characteristics. To gauge the presence of a life-threatening event, we also asked participants whether they had gone through a period of coma >1 h and/or whether they had stayed in intensive care. On this basis, each participant with a total score of ≥27/80 on the NDE-C scale was assigned to one of the two subgroups: a “classical NDE” group (i.e., experiences that occurred in a life-threatening context) or an “NDE-like” group (i.e., similar phenomenological experiences that occurred in a non-life-threatening context; [3]). The reported experiences were assessed according to the NDE-C scale. They were then asked to complete the EDI, the EII and the NR-6.

### 2.2. Measures

#### 2.2.1. The Near-Death-Experience Content (NDE-C) Scale

This tool is a 20-item self-report questionnaire used to identify the presence of a NDE phenomenology (i.e., cut-off score of ≥27/80; [7]). This scale can be divided into five factors covering dimensions related to *beyond the usual*, *harmony*, *insight*, *border*, and *gateway*. The factor *beyond the usual* includes speeded thoughts, an altered time perception, unusual and extrasensory perceptions, out-of-body experiences, and ineffability. The *harmony* subscale refers to positive feelings potentially experienced (i.e., well-being and harmony/unity). The *insight* subscale includes hearing voices without material incarnation, the feeling of suddenly understanding everything, precognitive visions, life review and encounters. The *border *factor refers to the sensation of leaving the earthly world, the feeling of non-existence/void/fear, coming to a point of no return, coming back from the experience as well as the feeling of dying. Finally, the *gateway* includes the vision or the entry in a bright light and/or a gateway. This multiple-choice scale enables to quantify the richness of the experience, with a total score ranging from 0 to 80. For each item, scores are arranged on a Likert format scale from 0 (0 = “not at all; none”; i.e., absence of the item) to 4 (1 = “slightly”, 2 = “moderately”, 3 = “strongly; equivalent in degree to any other strong experience”, and 4 = “extremely; more than any other time in my life and stronger than 3”; i.e., the presence of the item with a four-point gradation of intensity). Illustrative items are “*You felt a sense of harmony or unity, as if you belonged to a larger whole*” (item 6 assessing the experience of unity) and “*You had the impression of being outside of, or separated from your own body*” (item 11 assessing OBE). As suggested in a previous validation study, Martial et al. [7] indicate that it is the context within which the subjective experience has been precipitated that will permit the identification of the event as a “classical” NDE or an NDE-like episode.

#### 2.2.2. The Ego-Dissolution Inventory (EDI)

The EDI has been validated by Nour and his colleagues [3] and includes eight items related to the experience of ego dissolution (an illustrative item is “*I experienced a disintegration of my ‘self’ or ego*”). Participants are asked to give an estimate on a visual analog scale format from 0 to 100 (with incremental units of one), with 0 = “No, not more than usual” and 100 = “Yes, entirely or completely” (reflection of a maximal dissolution). The total score is the mean of the eight items. The higher the total score, the stronger the experience of ego dissolution.

#### 2.2.3. The Ego-Inflation Inventory (EII)

Nour and colleagues [3] also suggested eight other items to assess the experience of ego inflation. Those items are designed to reflect the distinct experience of unusually elevated self-assuredness and confidence. An illustrative item is “*My ego felt inflated*”. As for the eight items related to the ego dissolution, participants were asked to give an estimate on a visual analog scale format from 0 to 100 (with incremental units of one), with 0 = “No, not more than usual” and 100 = “Yes, entirely or completely”. The total score is the mean of the eight items. The higher the total score, the stronger the experience of ego inflation.

#### 2.2.4. The Nature-Relatedness Scale (NR-6)

The NR-6 scale is a short version of the *Nature-Relatedness Scale*. It consists of six items rated on a five-point scale (from 1 = “strongly disagree” to 5 = “strongly agree”), evaluating one’s level of self-identification and subjective sense of connectedness with nature [31]. Illustrative items are “*My relationship to nature is an important part of who I am*” and “*I always think about how my actions affect the environment*”. The total score is calculated by averaging the six items, whereby higher scores indicate a stronger connection with nature.

### 2.3. Statistics

Given the categorical nature of our data, McDonald’s ω was calculated for each questionnaire. The minimum acceptable value for McDonald’s ω is 0.70 and the maximum is 0.90 [32,33]. Below 0.70, the internal consistency is considered low, while a value above 0.90 indicates redundancy or duplication of items.

All variables were tested for normality (*p* < *0*.05) with the Shapiro–Wilk test. All distributions being skewed (except the NDE-C total score), data are reported as median (inter-quartile range; IQR) and non-parametric tests were used. Mann–Whitney U tests were performed to compare age at interview, age at NDE, and time since NDE between groups (classical NDE vs. NDE-like group). Pearson’s χ² tests were used to assess frequency distributions.

Group differences (classical NDE vs. NDE-like group) regarding all questionnaire total scores were evaluated using Bonferroni-corrected (*p* < 0.01) Mann–Whitney U tests. We also reported data as modal values for the EDI and EII.

We also compared the EDI and EII total scores of all NDE experiencers (i.e., including both classical NDE and NDE-like groups) using a paired-sample Wilcoxon signed-rank test.

We calculated Spearman rank-order correlations between each questionnaire’s total score for the whole group of NDE experiencers. Results were considered significant at *p* < 0.008 after Bonferroni correction.

Fisher’s r-to-z transformations were calculated to test any significant difference between the correlation coefficients between the NDE-C and the EDI total scores in the two groups of experiencers (i.e., classical NDE vs. NDE-like groups).

A linear regression was performed to determine the extent to which the scores at the EDI and the EII scales could explain the total scores obtained at the NDE-C.

Finally, we calculated Spearman rank-order correlations between the EDI total scores and two items of the NDE-C scale: item 11 (Out-of-body experience) and item 6 (Harmony/ Unity)

## 3. Results

### 3.1. Demographic Data

Out of 109 respondents, the final total sample consisted of 100 experiencers (i.e., meeting the criteria for NDEs: NDE-C scale total score ≥27/80; [7]) (“all NDE” group) who had completed all questionnaires: 61 females; median age at interview = 59 years, IQR 48–64; median age at NDE = 29 years, IQR 19–42. The sample consisted of (i) 80 classical NDEs (classical NDE group), including different near-death events: 13 anoxia (e.g., cardiac arrest), 24 traumas (e.g., car accident, falls), six complications of surgery or childbirth, and 37 other (non-traumatic events, such as hemorrhage or septic shock); and (ii) 20 NDE-like episodes (NDE-like group), including five during sleep, one during a meditative state, four during syncope, two during high anxiety, one during orgasm, and seven unknown causes (occurring spontaneously, cause not identified by the experiencer) (see Table 1 for demographic data).

### 3.2. Questionnaires

Table 2 presents the results of all the questionnaires for the whole sample and for the two groups. No significant differences were found between groups.

McDonald’s ω was deemed to be good for the EDI (ω = 0.88), the EII (ω = 0.82) and the NR-6 (ω = 0.90). The NDE-C showed an acceptable ω = 0.71.

**Figure 1:**
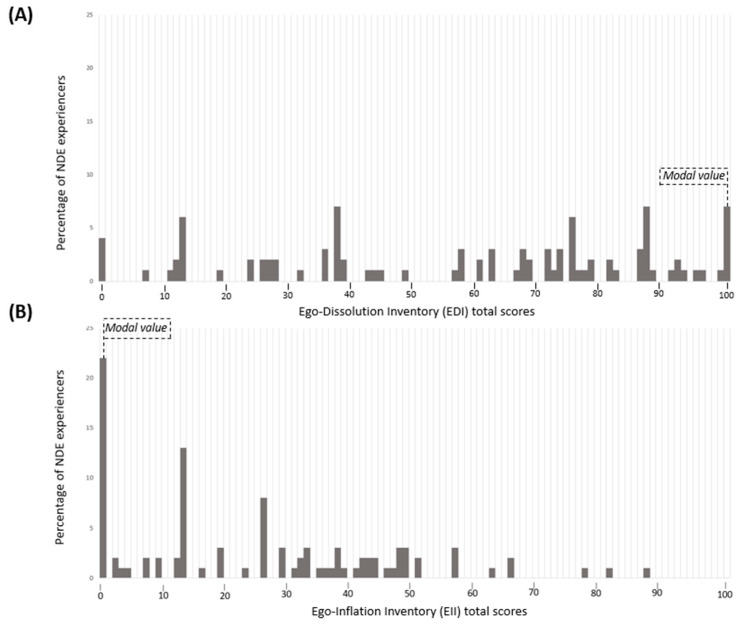
(**A**) The chart above refers to the frequency distribution of the experience of ego dissolution (EDI total scores) in all experiencers. Note that the only modal value is 100, even if the grouping of the different total scores in one single histogram bar visually pretends there are other modal values (same histogram bar heights for the bars at 38% and 88%). (**B**) The chart below refers to the experience of ego inflation (EII total scores) in all experiencers.

When looking at the whole group of NDE experiencers, we observed a modal value of 100 for the EDI total scores and of 0 for the EII total scores (see Figure 1). As can be observed in Figure 1, four experiencers (4%) did not experience any ego dissolution, as reported by a total score of 0 on the EDI. Interestingly, these four experiencers also rated 0 on the EII.

When looking at the classical NDE group only, we observed a modal value of 37.5 for the EDI total scores and of 0 for the EII total scores. For the NDE-like group only, we observed a modal value of 35 for the EDI total scores and of 0 for the EII total scores.

In addition, we observed higher EDI total scores compared with EII total scores (z = 4289; *p* < 0.001) among all NDE experiencers.

### 3.3. Correlations between Questionnaires

**Figure 2:**
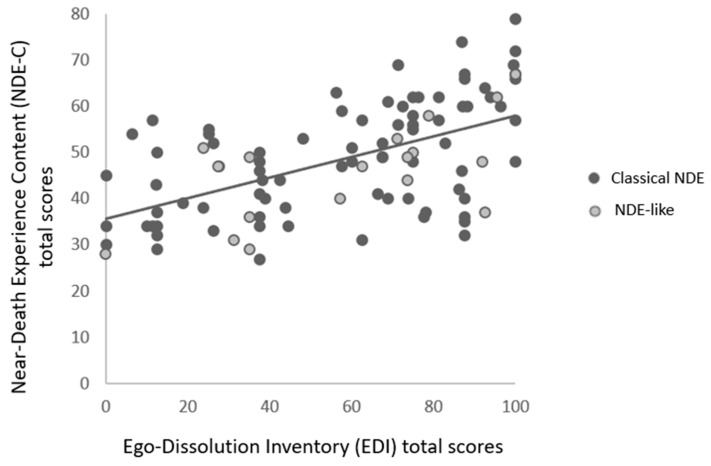
Association between the Near-Death-Experience Content (NDE-C) scale total scores and the Ego-Dissolution Inventory (EDI) total scores within the classical NDE and NDE-like experiencers (r = 0.55, IC_95_ = 0.41–0.68, *p* < 0.001).

**Figure 3:**
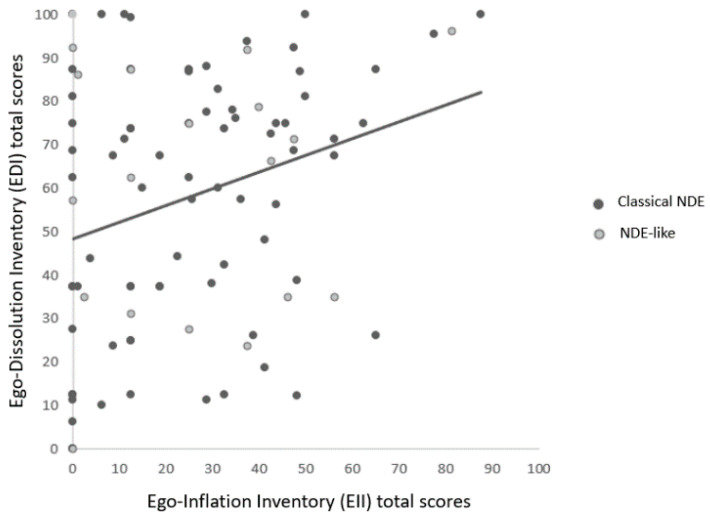
Association between the Ego-Dissolution Inventory (EDI) total scores and the Ego-Inflation Inventory (EII) total scores within the whole sample of experiencers (i.e., classical NDEs as well as NDE-like; r = 0.23, IC_95_ = 0.08–0.45, *p* = 0.021).

NDE-C scale total scores were positively correlated with EDI (see Figure 2), EII and NR-6 total scores (see Table 3). EDI total scores were positively correlated with NR-6 total scores. The overall linear model including the EDI, EII and NDE-C total scores was significant (F(2,97) = 26.7, *p* < 0.001; R^2^ = 0.35), thereby indicating that the two predictors explain 35.5% of the NDE-C total scores (EDI: estimate = 0.203, t = 5.99, *p* < 0.001; EII: estimate = 0.113, t = 2.38, *p* = 0.019). Figure 3 shows the individual scores of the whole sample on the EDI and EII. The Fisher’s z test showed that the two associations between the NDE-C and the EDI total scores of the classical NDE group (r = 0.58, IC_95_ = 0.42–0.72) vs. the NDE-like group (r = 0.49, IC_95_ = 0.15–0.80) were not significantly different (z = 0.47; *p* = 0.32).

OBE and a sense of unity (i.e., scoring above 0 on these items, thus reflecting the presence of the feature) are reported by 87% and 83% of the experiencers, respectively. Of note, out of the 13 participants who did not experience an OBE, 12 participants scored above 0 on the EDI total score and 9 scored above 0 on the EII total score. EDI total scores were positively correlated with the responses to item 11 (Out-of-body experience) of the NDE-C scale (r = 0.44, IC_95_ = 0.19–.53; *p* < 0.001) and with the responses to item 6 (Harmony/Unity) of the NDE-C scale (r = 0.44, IC_95_ = 0.25–0.58; *p* < 0.001).

## 4. Discussion

The present study sought to retrospectively explore the frequency and intensity of the experience of ego dissolution and inflation during NDEs. We observed higher EDI total scores compared with EII total scores in our sample of experiencers. Indeed, higher EDI total scores were more common than low EDI total scores, whereas it was the opposite trend for the EII: low EII total scores were more common than higher EII total scores. The results also revealed that higher scores of NDE ‘richness’ were associated with higher EDI total scores. In other words, the richness of the NDE was strongly (r = 0.55) associated with the subjective intensity of the experience of ego dissolution. Interestingly, the experience of ego dissolution retrospectively reported by our participants is rather intense on average (median of 67/100 on the EDI) and comparable to what is reported after ingestion of psychedelics (e.g., 46/100 reported during a DMT-induced psychedelic experience in [23]; 74/100 reported after a 5-MeO-DMT-induced psychedelic experience in [34]; 41/100 reported after lysergic acid diethylamide [LSD] in [3]; 52/100 reported after psilocybin intake in [35]). However, no direct comparison regarding the experience of ego dissolution between NDE and drug-induced psychedelic experiences has been carried out yet. As a matter of fact, it would be interesting to directly compare the sense of self experienced in NDEs and psychedelic experiences using a sample of people who experienced both types of experiences. Moreover, we observed that the intensity of the experience of ego dissolution was positively correlated with the sense of harmony/unity experienced during the NDE (r = 0.44), as if they belonged to a related construct or phenomenon. One can hypothesize that this feature may be experienced as an inevitable counterpart of the experience of ego dissolution; the blurring of personal boundaries could thus lead to feelings of belonging to a larger whole or ‘unity’, as discussed elsewhere [3,20].

Even though an intense experience of ego inflation was not commonly reported by our participants, the richness of the NDE was also correlated (albeit less strongly; r = 0.34) with the experience of ego inflation. Our study shows that NDE experiencers may score more than 0 (i.e., suggesting a minimum of disturbance of the sense as compared to their normally well-circumscribed experience of self in daily life) on both scales (the EDI and EII); however, we found higher EDI total scores compared with EII total scores in our participants’ sample. Nonetheless, our study does not permit to know more precisely when and how each type of ego experience has occurred within the NDE itself where many features are experienced. Although researchers from various fields have recently generated considerable interest in NDEs, the study of the sense of self in NDEs, in particular, has not received much attention yet. Further studies are necessary to explore this question.

OBEs are reported here by 87% of the sample of experiencers. This is consistent with the literature indicating that OBE is the second most commonly encountered feature in NDEs (i.e., about 80%; [6,8,9,10]). As expected, we found that the more intense the OBE, the more intense the experience of ego dissolution (r = 0.44). This raises questions about the link between the potential breakdown of the sense of self and the OBE, even if our study does not permit conclusions about any causality. The correlation between the two was likely and expected due to the close link between the two phenomena [12]. Nonetheless, it is worth mentioning that OBEs may be related to different types of disturbance of the sense of self. Indeed, we can dissociate three types of experience of self: (i) self-identification with a body (“*do I have a body? If so, what is my body*?”), (ii) self-location in space (“*where am I?”*), and (iii) the visuospatial perspective we experience (“*from where do I experience the world?”)* [12,36]. Future studies should address the experience of disruptions of the sense of self during OBEs, which is (spontaneous or induced by drugs or practice such as meditation) not an uncommon occurrence (prevalence of around 9 to 12% in surveys of the general population; e.g., [37,38]).

We observed that both groups of experiences did not differ in terms of richness of the experience. This is consistent with our previous studies (e.g., [6,7,39]). Moreover, no difference was observed between their experience of ego dissolution and inflation. Our results provide additional empirical evidence that classical NDEs and NDE-like episodes are not dissimilar. If this is confirmed, one may hypothesize that neurophysiological mechanisms underlying NDEs could be activated spontaneously or in non-life-threatening situations where the physical threat is only perceived and not necessarily actual [30,40]. Our results suggest that the circumstances of occurrence (i.e., in or outside the context of a life-threatening circumstance) does not influence reports of ego dissolution. Nevertheless, future studies studying the sense of self in a larger sample of NDE-like experiencers are warranted to confirm our findings.

We observed that the subjective connection with nature reported by experiencers was associated with both the richness of the NDE and the experience of ego dissolution (r = 0.27 and r = 0.41, respectively). Relatedly, Nour and colleagues [13] have already shown that experiences of ego dissolution under a psychedelic are dose-dependently associated with ratings of connectedness to nature, a finding which has also been replicated in surveys [28,41] and controlled research [40] showing that acute psychedelic experiences of ego dissolution predicted greater nature connectedness, including in a causal fashion [41,42]. Regarding the present results, we can also hypothesize that people who feel more connected to nature may be more prone to experience an NDE(-like) episode, notably due to some traits such as an “openness to experience” trait. In the present study, the NR-6 was only administered retrospectively to the NDE(-like) experience, which happened some months or years ago though, thereby limiting our conclusion regarding any causality between NDEs/NDE-like experiences and the subjective sense of connectedness with nature. Future studies are necessary to investigate the impact of NDEs on the construct of nature-relatedness.

The most frequently reported changes after a classical NDE correspond to a more altruistic and spiritual attitude, an important personal understanding of life and self, decreased fear of death, as well as a trend towards less materialist values [10,43,44,45,46,47,48,49]. Psychedelic studies show similar long-term changes, such as reduced death anxiety and lasting improvements in well-being [21,50,51,52,53,54]. While the overlap between NDEs and psychedelic experiences have been reported in terms of phenomenology (e.g., [23,30]), no direct comparison between NDE and drug-induced psychedelic experiences regarding their long-term psychological effects has been carried out yet. Interesting questions to investigate include: what are the underlying neurobiological mechanisms potentially linking the two experiences? Do NDEs and psychedelic states reflect closely related brain states albeit via different means of induction? The recently proposed ‘pivotal mental states’ model [55] bears relevance here. This article proposes that the experience of ego dissolution is a common feature of NDEs and (high-dose) psychedelic states, characterized by a quality of consciousness that is profoundly less ego-centric. Recent fMRI findings suggest that the degree of ego dissolution correlates with increased global functional connectivity in some brain areas such as the angular gyrus [56] and with default mode network integrity [57]. Although this is speculative, these patterns may be considered a good candidate for neural correlates of the experience of ego dissolution experienced during NDE(-like) as well. It is worth noting that this is mostly inconsistent with the phenomenon of ego inflation. Nonetheless, still very little is known about the sense of self that may characterize the different altered states of consciousness that humans can experience; a possibility is that both experiences could potentially emerge within a single subjective experience and consequently may not necessarily be antithetical.

To date, there have not been any systematic studies focused on the broader life and psychological impact of NDE-like encounters. Connectedness to nature and nature exposure are associated with improved mental health outcomes [31,41]; thus, it would be interesting to assess whether classical NDEs and/or NDE-like episodes are also associated with improved mental health [48]. NDE-like phenomenology may be reproducible in laboratory settings using various techniques (e.g., hypnosis, syncope through hyperventilation and orthostasis and Valsalva maneuver; [58,59]). If these lab models are faithful to the naturalistic phenomena, they could offer a useful means of assessing the longer-term psychological sequelae of NDEs and NDE-like episodes, e.g., as has been carried out with lucid dreaming (e.g., [60]) and psychedelic treatment (e.g., [50]).

Existing literature suggests that disturbance in the sense of the self should be viewed along a continuum [18]. Usually, healthy people have a constant and coherent sense of being an “embodied self” which encompasses our emotions, thoughts, and actions, and is generally felt to be distinct from other people and separate from the external environment [61,62]). However, anomalies to this principle do not necessarily indicate underlying psychopathology [61], and are even highly valued (e.g., as conducive to insight) in certain spiritual traditions. Moreover, such phenomena can be transiently induced via experimental means [63,64,65], and indeed, the psychedelic drug state can be considered one such example. Given the relevance of self-consciousness to our understanding of human consciousness in particular, investigating the nature of self-disturbances promises to offer fundamental insights into the nature of consciousness.

It is important to acknowledge the limitations of this study. First, volunteers enrolled in the study were self-selected and hence might not be representative of the broader population. Second, our study was cross-sectional and relied on short self-report measures. As our aim was to assess the sense of self, we relied on retrospective subjective measures of first-person experiences; only the individuals who experienced the NDEs know “what it feels like” to have had such experiences. As with psychedelic experiences [66], it is common for NDEs to be described as ‘ineffable’ [7,46]. Here, we decided to use a closed questionnaire format rather than spontaneous written narratives or freely expressed reports. The advantage of this approach is our ability to quantitatively assess the relevant phenomena using validated measures, but the limitation is that this constrains free expression and potential new learnings about the relevant phenomena. Third, our participants were Western, thus limiting generalizability to other populations. Fourth, it seems that there is a large difference in group sizes between NDE and NDE-like experiencers. Given the specificity of this population, the recruitment of participants was arduous and we were limited in our recruitment. Future studies should include a larger group of NDE-like experiencers. Other limitations are the lack of medical information regarding the presence of a life-threatening event and the lack of control groups or conditions. The EDI and EII are completed using a visual analog scale in which the zero is defined as “*No, not more than usually*”, thus allowing comparison with their usual experience of self. Moreover, we would like to mention that some experiencers of the present sample were also involved in other published studies from our research group due to the relative scarcity of NDEs and the challenge to recruit NDE experiencers. Finally, the retrospective and correlational design of our study does not permit conclusions to be made about any causal pathway. Hence, the present results need to be interpreted cautiously.

## 5. Conclusions

This study suggests that the experience of ego dissolution is a common feature of NDEs. The richness of the NDE was positively correlated with the intensity of this experience of ego dissolution and, less strongly, with the intensity of the experience of ego inflation and subjective connection with nature. In addition, we observed a positive correlation between EDI total scores and the intensity of the OBE and the sense of unity. Overall, our results are in line with existing literature suggesting that mystical-type experiences, including NDEs, may feature important disturbances in ego-boundaries and highlight the fact that NDEs in particular can be considered as a probe to explore human (self) consciousness.

## Figures and Tables

**Table 1:** Comparison of demographic data in the classical NDE and in the NDE-like subgroups (Pearson’s χ² test was used for “gender” and Mann–Whitney U tests for other variables).

Demographics	Classical NDE *n* = 80	NDE-Like *n* = 20	*p*	Effect Size
Gender—female	47 (59%)	14 (70%)	0.36	-
Age at interview *Median (IQR)*	60 (49–65)	56 (46–61)	0.45	0.109
Age at NDE *Median (IQR)*	30 (19–43)	26 (17–39)	0.36	0.132
Time since NDE (year) *Median (IQR)*	27 (9–38)	26 (11–35)	0.92	0.013

**Table 2:** Total scores of all questionnaires for the whole sample, and for the two subgroups (comparisons between subgroups were performed using Mann–Whitney U tests).

Questionnaires (Min–Max Total Score)	All NDE *n* = 100	Group		*p* Value	Effect Size
		Classical NDE *n* = 80	NDE-Like *n* = 20		
NDE-C total score (0–80) *Median (IQR)*	48 (38–57)	49 (38–59)	47 (39–50)	0.177	0.196
EDI (0–100) *Median (IQR)*	67 (35–84)	65 (35–82)	69 (35–87)	0.629	0.071
EII (0–100) *Median (IQR)*	24 (3–40)	21 (6–39)	25 (2–41)	0.855	0.027
NR-6 (0–5) *Median (IQR)*	4 (4–5)	4 (4–5)	4 (4–4)	0.716	0.053

**Table 3:** Spearman rank correlations between total scores of all questionnaires (*n* = 100). * *p* < 0.008; ** *p* < 0.001.

	NDE-C Scale	EDI	EII
EDI	0.55 ** (IC_95_ = 0.41–0.68)		
EII	0.34 ** (IC_95_ = 0.16–0.50)	0.23 (IC_95_ = 0.08–0.45)	
NR-6	0.27 * (IC_95_ = 0.07–0.44)	0.41 ** (IC_95_ = 0.23–0.56)	0.09 (IC_95_ = 0.07–0.31)

## Data Availability

Some or all data used during the study are available from the corresponding author by request.
